# A Combined Study on Optimization, *In Silico* Modeling, and Genetic Modification of Large Scale Microbial Cellulase Production

**DOI:** 10.1155/2022/4598937

**Published:** 2022-12-21

**Authors:** Md. Raisul Islam Rabby, Zabed Bin Ahmed, Gobindo Kumar Paul, Nafisa Nusrat Chowdhury, Fatema Akter, Mamudul Hasan Razu, Pranab Karmaker, Mala Khan

**Affiliations:** Bangladesh Reference Institute for Chemical Measurements, Dhaka, Bangladesh

## Abstract

Cellulase is a biocatalyst that hydrolyzes cellulosic biomass and is considered a major group of industrial enzymes for its applications. Extensive work has been done on microbial cellulase but fungi are considered a novel strain for their maximum cellulase production. Production cost and novel microbial strains are major challenges for its improvement where cheap agro wastes can be essential sources of cellulose as substrates. The researcher searches for more cellulolytic microbes from natural sources but the production level of isolated strains is comparatively low. So genetic modification or mutation can be employed for large-scale cellulase production before optimization. After genetic modification than *in silico* molecular modeling can be evaluated for substrate molecule's binding affinity. In this review, we focus not only on the conventional methods of cellulase production but also on modern biotechnological approaches applied to cellulase production by a sequential study on common cellulase-producing microbes, modified microbes, culture media, carbon sources, substrate pretreatment process, and the importance of optimum pH and temperature on fermentation. In this review, we also compare different cellulase activity determination methods. As a result, this review provides insights into the interrelationship between the characteristics of optimizing different culture conditions, genetic modification, and *in silico* enzyme modeling for the production of cellulase enzymes, which may aid in the advancement of large-scale integrated enzyme manufacturing of substrate-specific enzymes.

## 1. Introduction

The planet's most abundant biomass is cellulose, a linear polysaccharide of D-glucose subunits. This cellulosic polymer creates 1, 4-glycosidic linkages between individual glucose residues [1] and a primary component of the plant cell wall [2]. Cellulase is an enzyme family that hydrolyzes cellulose [3], also known as carbohydrate-active enzymes (CAZyme) [4], with biotechnological potential in a variety of industries including food, textile, animal feed, brewing, agriculture, biomass refining, pulp, and paper [5–8]. It occupies the third most significant industrial enzyme on the worldwide market (i.e., ≈15%) after amylase (≈25%) and protease (≈18%). Cellulase enzymes are classified into three types: endoglucanase (endo-1, 4-D-glucanase, EG, and EC three.2.1.4); exoglucanase (exo-1, 4--D-glucanase, CBH, and EC three.2.1.91); -glucosidase (1, 4--D-glucanase, BG, and EC three.2.1 [9, 10]. Their high production cost and low yielding capacity are the major problems for industrial applications [11], but an effective and profitable enzymatic hydrolysis process must be economical [12]. Renewable carbon sources and noble microorganisms are major contributors to cellulase production [13]. The lignocellulosic materials, for example, wood, waste paper, corn cob, wheat bran, waste paper, sludge [12, 14], sugar cane bagasse [15], wheat straw [16–18], aspen wood, willow [19], and waste newspaper [20, 21] are effective carbon sources for this enzyme. So cheap biomass resources may significantly serve cellulase production, decreasing production prices [22].

Enzymes are mostly produced by microorganisms that can be cultured in large quantities within a short period [23]. So the use of eco-friendly microorganisms for lignocellulosic material pretreatment is currently gaining much attention in the industry [24]. Bacteria, fungi, and actinomycetes are capable of hydrolyzing cellulosic materials. The kingdom fungi include the genus like *Aspergillus*, *Penicillium*, *Chaetomium*, *Trichoderma*, *Fusarium*, and *Alternaria*. [25] Cellulolytic bacteria include *Cellulomonas*, *Cellvibrio*, *Pseudomonas* sp. *Bacillus*, and *Micrococcus* [26, 27]. Fungi are energetic decomposers and are probably responsible for 80% of the polysaccharide breakdown in the world [28]. So, these fungi can be the preferred source of cellulase for commercial purposes because they release large amounts of cellulase into the culture medium. Although there are a significant number of fungi that generate cellulase enzymes, only a handful have been thoroughly examined since they produce considerable amounts of these extracellular enzymes [29]. The fungal cellulases are less complex extracellular that used to be more rapidly cloned, whereas *Trichoderma reesei* is a commonly cited mesophilic filamentous Ascomycota fungus [30] and its industrial enzyme titers above 100 g/l [31]. To increase the production of enzymes and cellulose hydrolysis, it is crucial to modify the strains through random mutagenesis. Heavy ion irradiation has been effectively employed for the mutation breeding of microorganisms to develop novel strains with industrial application potential and produced a significant number of outstanding mutants [32]. Solid-state fermentation, Batch fermentation, and Submerged fermentation were applied for the production of cellulase enzyme [33–35]. Solid-state fermentation (SSF) is gaining popularity as a cost-effective and equally valuable method for the bioconversion of lignocellulosic material utilizing cellulolytic bacteria [36, 37]. In microbial cultures, cellulase production is strongly reliant on growth, and several variables impact productivity [38]. The key deciding parameters for cellulase synthesis are believed to include carbon and nitrogen supplies, temperature, pH, and dissolved oxygen in liquid broth [39, 40]. With several applications in protein therapies, biocatalysts, bioengineering, and other biomedical fields, enzyme design is a significant area of active research [41]. Experimental and computational methodologies can be combined to produce more effective industrial enzymes by amplifying and completing experimental results [42]. For this enzyme class, however, we only have a limited grasp of their structure, dynamics, and enzymatic function. So this review highlights the potential utilization of microorganisms for cellulase production, strain improvement by mutagenesis to enhance enzyme production, molecular modeling, factors affecting enzyme production, and its application in different industries.

## 2. CAZy Database and Cellulase Involved in CAZymes

All enzymes engaged in the alterations, breakdown, or biosynthesis of carbohydrates and their derivatives are referred to as carbohydrate-active enzymes (CAZymes) [43]. After 25 years of continuous research, the classification of carbohydrate-active enzymes (CAZymes) is now divided into several hundred distinct enzyme protein families [44]. All known CAZymes are categorized by the CAZy database and related bioinformatics tools into the following classes: glycosyl transferases (GTs), polysaccharide lyases (PLs), carbohydrate esterases (CEs), glycoside hydrolases (GHs), and auxiliary activities (AAs) [44, 45]. Lignocellulosic plant biomass can be broken down into simple sugars and then transformed into biofuels and other products by the use of CAZymes such as cellulases and xylanases [46]. In several sectors, CAZymes produced by microorganisms, particularly fungi, are employed. Finding the best candidate for a fungus, however, is an expensive and time-consuming process. In this regard, the “CAZymes Based Ranking of Fungi (CBRF)” web database has been created for sorting and choosing an optimum fungal candidate based on their genome-wide distribution of CAZymes [47]. The present CAZy database, which mostly lists catalytic domains of carbohydrates-active enzymes, is related physically (CAZymes). It was first developed in 1991 as a categorization for glycoside hydrolases (GH), and at the moment, this component of CAZy accounts for the majority of it, with 172 GH families [48]. Maintaining and updating the family categorization of this class of enzymes, classifying freshly available sequences from GenBank and the Protein Data Bank, and capturing and presenting functional information for each family are the three main responsibilities of the CAZy curators [49].

## 3. Common Cellulolytic Microbes

Cellulolytic microbes primarily destroy carbohydrates and are unable to use lipids and proteins as energy sources for metabolism and development. A wide range of carbohydrates may be used to make cellulases by a variety of microorganisms. In suitable fermentation circumstances, bacteria can create cellulase enzymes by breaking down cellulosic materials [50].

**Figure 1:**
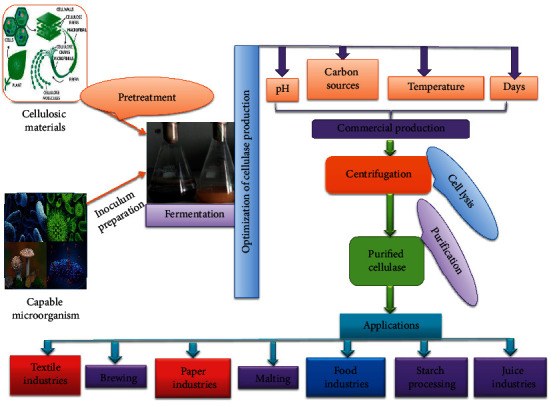
Diagrammatic representation of microbial cellulase production and its industrial applications.

These microorganisms indicated fungi, bacteria, and actinomycetes groups. Mawadza et al., and Wood [51, 52] reported that aerobic bacterial species like *Cytophaga*, *Cellulomonas,* and *Cellovibrio* can degrade cellulosic materials and produce this crucial enzyme, whereas some other studies reported that the efficient cellulase-producing fungi species including *Trichoderma*, *Penicillium*, *Fusarium*, *Alternaria, Aspergillus*, and *Cladosporium*. The fungi are responsible for 80% breakdown of cellulose, whereas cellulase-producing fungi are subdivided into two groups such as aerobic and anaerobic fungi [53]. The adaptive nature and extracellular characteristics of aerobic fungi are generally ideal for producing most of the cellulases used in industry [54]. *Trichoderma reesei* is the most extensively researched fungus and can convert both wanted and native cellulose to glucose. Due to researchers suggested that the maximum expensively intentional aerobic fungus is *T. reesei* which has the highest ability to hydrolyze local cellulose [55, 56] and other microbes. The previously reported cellulase-producing fungi, bacteria, and actinomycetes are given in Table 1, and a common method of microbial cellulase producing given in Figure 1. However, strains that have undergone genetic modification are capable of producing cellulase in comparatively greater quantities [37].

## 4. Genetically Modified Microbes

Since 1990, genetically modified microbes were used in industrial production. A good strain is selected based on targeted physiological properties and functionality which should be high product yield capable and resistant to environmental stress [82]. Overexpression of the cellulase gene has been achieved by a variety of genetic approaches. Various microbial strains such as *Trichoderma reesei, Saccharomyces cerevisiae*, and *Bacillus subtilis* have been genetically modified for gene expression. When modified *L. plantarum* was cultured in a bioreactor its cellulolytic activity was 33.4 U/mg. *T. reesei* was randomly altered at Rutgers University, resulting in the strain RUT-C30, which demonstrated a 20-fold increase in cellulase secretion. According to Adsul et al., [83], mutant *T. reesei* RUT-C30 is one of the most widely used fungal strains for commercial cellulase production. *Bacillus pumilus* was randomly altered, resulting in cellulase yields four times greater than the wild-type strain [84]. The *Aspergillus* was subjected to irradiation of Co60 and UV treatments. *Aspergillus* sp. XTG-4 mutant generated 19 times more than the wild-type strain [85]. Although the fungus *Macrophomina phaseolina* generated EG, site-directed mutagenesis was used to create enzymes that needed novel substrates by modifying conserved sections of this enzyme family [86]. Genetic engineering can be used to manipulate microorganisms for the production of high metabolites, but due to the inherent complexity of the organism, it may not be as simple as one might think. Nakari–Setälä et al. [87], reported that cre1 was eliminated or replaced by increased enzyme production and may serve as an effective target gene in manipulating *T. reesei* to enhance enzyme production.

## 5. Molecular Modeling

**Figure 2:**
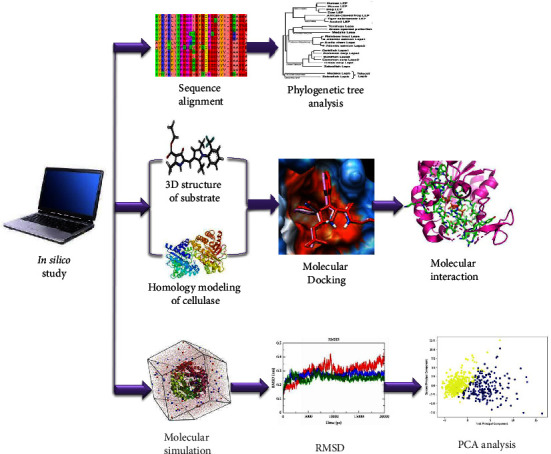
*In silico*-based study on microbial cellulase enzyme for the understanding of its different properties.

Currently, researchers are focusing on the bulk production of industrially relevant enzymes with significant biotechnological applications using various *in silico* methodologies such as docking, molecular dynamics simulation, protein modeling, genetic engineering, metagenomics, and protein engineering on cellulase enzymes [88]. The current study focuses on computer-assisted modeling, which is a vital strategy for evaluating a small molecule's binding affinity at the binding site of its macromolecular target. The protein-ligand interaction is the most exciting example due to its industrial applications. The energy scoring function is used to score the ligands based on the protein structure between them, and the posture with the lowest energy score is deemed the best match. Selvam et al. [89], reported the binding efficiency of the *Acinetobacter* cellulase enzyme. The binding energies of the four polysaccharide subunits, cellobiose, cellotetraose, cellotetriose, and laminaribiose, are −6.15 kJ/mol, −7.88 kJ/mol, −6.16 kJ/mol, and −6.6.72 kJ/mol, respectively. These docking studies showed that cellulase has a higher potential than cellotetraose as a substrate for high yields of ethanol. Hoda et al. [90], an *in silico* structure, function, and phylogenetic analysis of cellulase from the bacterium *Ruminococcus albus* was performed. They obtained the *R. albus* cellulase protein sequence from the UniProt database and the 3D structure was predicted by homology modeling. Tamboli et al. [91], *in silico* physicochemical analysis of cellulase enzymes of the fungi *Trichoderma* and *Aspergillus* were performed. Their study found that the content of secondary structures such as alpha helices and random coils predominates in the 3D conformation of these fungal cellulases. According to the molecular docking study conducted in their study, *A. Niger* cellulase residues Glu160, Trp200, and Thr201, and *T. Longibrachiatum* Tyr168, Tyr192, Gln196, and Asp220 were found to be involved in the interaction with substrate cellulose. In their study, Lugani, 2017 published various *Bacillus* sp. The amino acid sequence of cellulase was also analyzed [92]. The catalytic reaction depends on the structure of the enzyme. Molecular dynamics is an important method for determining the dynamics of protein structure, especially the loops or domains involved in the catalytic activity of enzymes. Paul et al. [93], studied the structural properties of various microbial cellulases based on the structures predicted by molecular modeling methods. They also used molecular docking between receptor proteins and ligands to present molecular interactions with substrate molecules and their networks. To compare the catalytic activity of wild-type and mutant enzymes developed using *in silico* technology, the bond energy between the enzyme and the substrate was computed. Their research suggests that cellulose hydrolysis can be improved for larger bioethanol outputs. Ali et al. [94], also found that uncovering Cel6A variations from *Thermobifida fusca* utilizing protein domain engineering and molecular dynamics investigations improved their enzymatic activity. Computer-based different microbial cellulase enzyme is given in Figure 2.

## 6. Microbial Culture Media Preparation

Media is a primary factor for microbial growth and enzyme production. Most of the research suggested that Potato Dextrose Agar (PDA) and Sabouraud Dextrose Agar (SDA) are used as common fungal culture media, whereas LB broth and LB agar media were used for primarily bacterial culture preparation. The Mandel and Weber media established a cellulolytic fungi enzyme production medium which is still used for cellulase production [95]. The Mandel's and Weber media contains tween 80, (NH_4_)_2_SO_4_, K_2_HPO_4_, MgSO_4_·7H_2_O, and the optimum pH was 4.8. The medium's carbon source is microcrystalline cellulose, which contains various salts as microelements. Iqbal et al. [96], reported that Vogel's nutrient medium was used for inoculum preparation of fungi under SSF. Thus, studies focused on inoculum media optimum compositions [14, 97–104] as well as the nutrition, pH, temperature, and incubation times are essential for inoculum growth and microbial fermentation [14, 105, 106].

## 7. Substrates and Pretreatment Process

**Figure 3:**
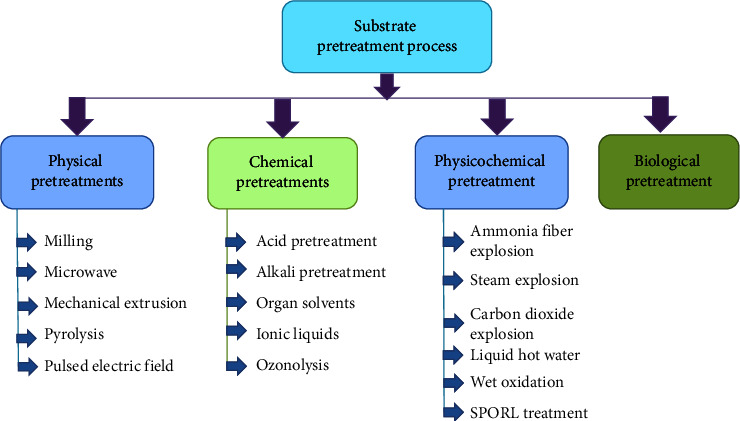
Different processes of the substrate pretreatment.

Cellulosic materials are the main component of cellulose, whereas lignocellulose biomass is an inexpensive source for cellulase production [54]. These materials indicate as sugarcane bagasse, aspen wood, wheat straw, and corn cobs, are economical sources of carbon for cellulase production. Liming and Xueliang [12] reported that corn cobs are used as a residue for cellulase production that can efficiently be utilized by the fungus. Weeds can also be a low-cost substrate as it grows naturally and is available in nature, whereas vegetable fibers can be used as a renewable source for cellulase enzyme [107]. Peels from *Luffa cylindrica* and *Litchi chinensis* have also been used for cellulase production [108]. Before, using these substrates as energy source pretreatment was necessary to improve enzyme hydrolysis rate and increase yields of fermentable sugars [109]. Pretreatment changes cellulosic biomass structures and increases the availability of cellulase enzymes. There are four types of substrate pretreatment processes used such as physical, chemical, physicochemical, and biological pretreatment processes (Figure 3). In the physical method, the surface, area, and pore size of lignocellulosic biomass are increased, but the polymerization and crystallinity of cellulose are decreased [109]. Chemical pretreatment is a less attractive method where chemical materials such as sulfuric acids, hydrochloric acid, ammonium, sodium, calcium, potassium, methanol, acetone, ethanol, ethylene glycol, and chloride are used. In the physiochemical method, high equipment and temperature are needed with ammonia fiber, steam, carbon dioxide, and SPORL. These conventional methods required high energy, nonpolluting equipment, and expensive reagents but biological pretreatment is environmentally friendly and consumes less energy where required living microorganisms such as fungi genera *Pleurotus*, *Ceriporiopsis*, *Ceriporia*, *Pycnoporus*, *Cyathus*, and *Basidiomycetes* [110].

## 8. Fermentation

Fermentation is a crucial step of enzyme production that is strongly influenced by different chemical compositions and chemical changes in the organic substrate through the activity of microorganisms [101]. In fermentation, substrate mass, heat, and oxygen transport are essential for microbial growth and enzyme production [103, 105]. Submerged fermentation (SmF) and solid-state fermentation (SSF) are two important forms of fermentation, according to Saqib et al. [111]. SmF involves microbial culture in the liquid medium for the synthesis of desired products, such as amylases and proteases [112].

SmF procedures are easily automated and do not suffer from heat mass transfer. According to Babbar and Oberoi [113], this approach has significant limitations because of the medium's high manufacturing cost and complexity. Solid-state fermentation (SSF) is a competitive technology for cellulase production because it has several benefits such as high productivity, relatively high product concentrations, improved monitoring, handling, and a less wealthy generation [114]. According to Tengerdy and Szakacs [115], the cost of producing cellulase in SSF is tenfold lower than in SmF, whereas John et al. [116], describe SSF as having direct importance to industrial enzymes and their direct agro-biotechnological applications as silage or feed additive, lignocellulosic hydrolysis, and natural fiber processing. *Theroascus aurantiacus* also generated xylanase and CMCase on SSF in various residues, according to Silva et al. [117].

## 9. Optimization of Parameters

### 9.1. Carbon and Nitrogen Sources

The researchers suggested that a large amount of cellulase production depends on a broad range of carbon sources [14, 118, 119]. González et al. [120], reported that carbon sources are not only an energy source for microorganisms but also an essential inducer for cellulase production and different carbon sources are disparity growth of an organism in different media [121]. Tangnu et al. [122], reported carbon sources to regulate the production of cellulase in fungi, where cellobiose, lactose, and sophorose are effective carbon sources. Cheng et al. [124] and Bhat and Bhat [125] reported that the highest cellulase production was obtained on cellulose-containing carbon sources. According to Margolles–Clark et al. [126], sugar, glucose, fructose, dextrose, and carboxy methyl cellulose were used to affect cellulase production in microorganisms, and dextrose is the best carbon source for fungi. Sophorose is a potent inducer of cellulase expression, whereas sophorose in the medium by trans-glycosylation could be the reason for the high levels of cellulase expression [127].

### 9.2. Optimization of pH

pH is the most influential factor affecting the microbial community to produce enzymes and strongly influences microbial growth [128, 129]. Firestone et al. [130], reported pH effects on multiple parameters and changed several factors that are hard to separate. Many studies focused on optimizing the pH, which is an important factor for fungal growth and enzyme production [131]. As a result, much effort has been expended in attempting to maximize cellulase production through optimal pH [132, 133]. The biggest issue during cellulase enzyme synthesis by diverse strains is controlling the pH of the medium. Prasetyo et al. [134], found that *A. cellulolyticus* has an ideal pH range for glucosidase of 5.5–6.0 and endoglucanase of 4.0, however Tangnu et al. [122], reported cellulase production by microorganisms in the pH range of 4.0–6.0. *T. reesei*, on the other hand, increased glucosidase enzyme synthesis when the pH was kept at 6.0. Hendy et al. [135], on the other hand, found a considerable reduction of cellulase synthesis when fermentation was undertaken at pH 5.0. These findings suggest that the ideal pH conditions for their performance vary among species. As a result, a technique for precise pH control based on the properties of individual cellulase components must be developed, and a targeted strain is required to increase overall cellulase production.

### 9.3. Optimization of Temperature

Enzyme production depends on different parameters; optimum temperature is one of them that influences enzyme productivity. Rojey and Monot [136] reported that optimum temperature is one of the most significant factors for cellulase enzyme production. Silva et al. [137], also reported cellulase production by microorganisms was determined from 30°C to 80°C range, with the highest production obtained at temperatures 30°C–40°C. When dairy manure is used as a medium, the highest cellulose production is at 25.5°C. Mutant *T. reesei* RUT-C30 produced the highest cellulase at a temperature of 30°C under solid-state fermentation [138], while *T. reesei* HY07, isolated from corn stalk, produced cellulase at 30°C [139].

### 9.4. Optimization of Incubation Day and Time

Nathan et al. [140], reported that enzyme production by the fungi started after 24 hours and the activities reached maximal levels within five to seven days of incubation. Acharya et al. [141], reported maximum cellulase production by *Aspergillus Niger* occurred after five days of fermentation, whereas *Trichoderma reesei* after six days in solid-state fermentation [142]. Darabzadeh et al. [143], reported that cellulase activity was higher in three days compared to six days.

## 10. Cellulase Activity Assay

**Figure 4:**
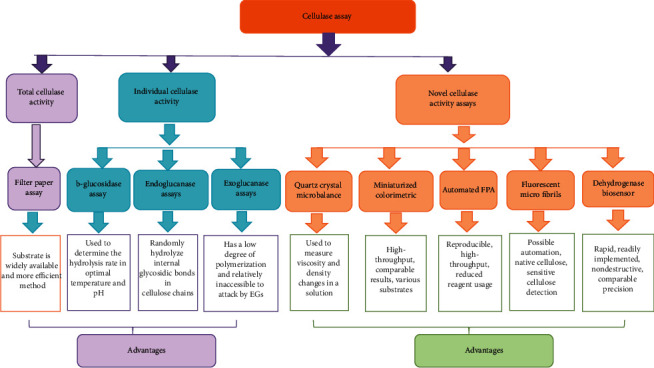
Different types of cellulase assay and advantages.

The cellulase activity determination methods are including the thread cutting [144] method, filter paper collapsing method [145], a spectrophotometric method [146], flat band method [147], branch and swain method [148], CMC method [149], and cellulase activity liquefaction method [150]. But Shuangqi et al. [151], reported that most new methods are used to determine cellulase activity via the DNS principle. Different cellulase assays are given in Figure 4.

## 11. Applications

Cellulase has been used in different industries for more than 30 years, such as pulp, paper, textile, bioethanol, wine, brewery, food processing, animal feed, agricultural, carotenoid extraction, detergent, and waste management. These industrial application sites are described in Table 2 and Figure 1.

## 12. Conclusion

The uses of cellulase in textiles are increasing day by day. This enzyme is eco-friendly and has no pernicious effect on the environment. Biotechnological applications of cellulases make prospects for the hyper-production of cellulases by genetically modifying fungal and bacterial strains. In the future, thermo-stable, alkaline-resistant cellulases will be made for industrial applications to attain high degradable yields. As Cellulase enzyme has applications in different industries, a bulk level of enzyme production is necessary. Before, bulk processing optimization of different parameters was vital as it affected microbial growth and production level. The world is dependent upon chemicals that negatively affect the ecosystem. Though lignocellulosic biomass is available, the pretreatment and production process is somewhat costly. So scientists are finding the cheapest way to produce cellulase enzymes to protect the environment and humankind.

## Figures and Tables

**Table 1:** Name of cellulase-producing microorganisms.

Group	Genus	Species	References
Fungi	*Trichoderma*	*T. reesei, T. branchiatum, T. viride, T. koningii, T. longibrachiatum, T. harzianum, T. atroviride*	[12, 37]
	*Aspergillus*	*A. niger, A. nidulans,* A. fumigatus, *A. oryzae, A. oryzae, A. terreus*	[57–61]
	*Fusarium*	F. solani, *F. oxysporum*	[62, 63]
	*Humicola*	*H. insolens, H. grisea*	[64]
	*Penicillium*	*P. brasilianum, P. occitanis, P. decumbans, P. funiculosum*	[65, 66]
	Others	*S. rolfsii, I.* lacteus, *A.* aculeatus, *S.* cellulophilum, *A.* cellulolyticus, *M.* albomyces	[67–69]
Bacteria	*Acinetobacter*	*A. junii, A. anitratus*	[70]
	*Bacillus*	*B. subtilis, B. pumilus, B. amyloliquefaciens, B. licheniformis, B. circulan, B. flexus*	[71, 72]
	*Clostridium*	*C. thermocellum, C. cellulolyticum, C. acetobutylium, C. papyrosolvens*	[73]
	Others	*A. cellulolyticus, Anoxybacillus sp., P. cellulose, T. fusca, A. cellulolyticus, R. marinus, R. albus*	[74–77],
Actinomycetes	*Cellulomonas*	*C. fimi, C.biazotea, C. uda*	[78]
	*Streptomyces*	*S. drozdowiczii, S. lividans,*	[79, 80]
	*Thermomonospora*	*T. fusca, T. curvata*	[81]

**Table 2:** Different industrial applications of cellulase enzymes.

Industry	Applications	References
Textile and detergent	Bio stoning of jeans and cotton, bio finishing of textiles, softening and enhancing garments' brightness, and removing dirt from cotton	[26, 152–154],
Paper and pulp	Dewatering, bio bleaching, enzymatic deinking of papers, bio pulping, bio characterization of pulp fibers, drainage difficulties reduction, and enhancing the hand sheet strength of the fibers	[155]
Food	Improve the quality of bakery products, flavor, viscosity, and clarification of juice, wine's aroma, and beer's filtration, an antioxidant such as carotenoid extraction, lower rancidity, fruits tenderization, reduction of roughage in doughs, and food preservation	[8, 148, 156, 157]
Agriculture	Weed control, control plant diseases, extent growth; improve the fertility of crops, plant cell wall breakdown, and production of cellulose from agricultural wastes	[6, 26]
Waste management	Municipal solid wastes (MSW) are used as an energy source for fermentation, hydrolyzing lignocellulose, sludge hydrolysis, bioconversion of agricultural wastes, bioethanol, and biofuel production	[11, 158–162]
Fermentation	Enhance malting and mashing, improve aroma quality of wine, and enhance viscosity, clarification, and filterability of juice and wine	[26]
Animal feed	Used as feed additives, improve nutritional value, improves animal health, improve meat quality, and improve silage production	[156, 163, 164]
Pharmaceutical and medical sciences	Inhibit biofilm formation in medical implants product containing cellulaselike digestion is essential for health—antibacterial chit oligosaccharides can be used as antitumor agents	[165–167]

## Data Availability

The datasets used and/or analyzed during the current investigation are accessible upon reasonable request from the corresponding author.
